# Per- and Polyfluoroalkyl Substances in Human Serum Samples of Selected Populations from Ghana

**DOI:** 10.3390/ijerph18041581

**Published:** 2021-02-08

**Authors:** Emmanuel Dartey, Dag G. Ellingsen, Balazs Berlinger, Yngvar Thomassen, Jon Ø. Odland, Jan Brox, Vincent K. Nartey, Francis A. Yeboah, Sandra Huber

**Affiliations:** 1 Faculty of Science and Environment Education, University of Education, Winneba, Mampong-Ashanti AM-0030-2291, Ghana; 2 National Institute of Occupational Health, N-0363 Oslo, Norway; balazs.berlinger@stami.no; 3 Department of Community Medicine, NTNU, The Norwegian University of Science and Technology, N-7491 Trondheim, Norway; jon.o.odland@ntnu.no; 4 Department of Laboratory Medicine, University Hospital of North Norway, N-9038 Tromsø, Norway; Jan.Brox@unn.no (J.B.); Sandra.Huber@unn.no (S.H.); 5 Department of Chemistry, University of Ghana, Legon, Accra GA-490-6862, Ghana; vknartey@gmail.com; 6 Department of Molecular Medicine, School of Medical Sciences, Kwame Nkrumah, University of Science and Technology, Kumasi AK-448-9252, Ghana; drfay180@gmail.com

**Keywords:** PFAS, occupational exposure, Ghana, human serum, blood mercury

## Abstract

The aims of this study were to assess serum concentrations of per- and polyfluoroalkyl substances (PFASs) in selected populations from Ghana, including workers engaged in the repair of electronic equipment (ERWs), and to elucidate PFAS concentrations in relation to blood mercury concentrations (B-Hg) as a biomarker of seafood consumption. In all, 219 participants were recruited into the study, of which 26 were women and 64 were ERWs. Overall, the PFAS concentrations were low. The most abundant components were perfluorooctane sulfonate (PFOS) and perfluorohexane sulfonic acid (PFHxS). Women had generally lower PFAS concentration than men. The ERWs had statistically significantly higher concentrations of perfluorooctanoate (PFOA), which was associated with the concentration of tin in urine. This could indicate exposure during soldering. The concentration of B-Hg was associated with several of the PFASs such as PFOA, PFOS and perfluoroheptane sulfonate (PFHpS). Additionally, the concentrations of perfluorodecanoic acid (PFDA) and perfluoroundecanoate (PFUnDA) were highly associated with the concentrations of B-Hg. It is noteworthy that the linear isomer of PFHxS was strongly associated with B-Hg while the branched isomers of PFHxS were not. In conclusion, the PFAS concentrations observed in the present study are low compared to other populations previously investigated, which also reflects a lower PFAS exposure within the Ghanaian cohorts. ERWs had significantly higher PFOA concentrations than the other participants. Several PFASs were associated with B-Hg, indicating that seafood consumption may be a source of PFAS exposure.

## 1. Introduction

Per- and polyfluoroalkyl substances (PFASs) belong to a group of chemicals with unique physico-chemical properties, having lipo- and hydrophobicity at the same time. The two main production methods are electrochemical fluorination (ECF) and telomerisation, resulting in different percentages of linear and branched PFASs [1]. These chemicals have broad and versatile applications [2], and some estimates indicate that more than 3000 PFASs have been used in industry and consumer products since being marketed in 1949, followed by an expanding number of applications [3]. They have been applied, e.g., in formulas for impregnation agents, firefighting foams, food contact papers and cookware, cosmetics, personal care products, ski waxes, stain and water repellant fabrics and a range of applications in, e.g., photographic imaging, aerospace/aviation, semiconductor, automotive and electronic surface coatings [2,3,4,5].

Scientific concern for PFASs has risen due to their global distribution and detection in the environment and humans. Among PFASs, perfluoroalkyl sulfonates (PFSAs) and perfluoroalkyl carboxylates (PFCAs) are resistant to environmental and biological degradation. Some analogs of these chemicals show bioaccumulative, biomagnification and toxic properties [6,7,8]. The most investigated PFASs, namely, perfluorooctane sulfonate (PFOS) and perfluorooctanoate (PFOA), have been associated with immune, metabolic and endocrine effects, while PFOA has been classified as a possible human carcinogen by the International Agency for Research on Cancer [9]. The usage of PFOS has been restricted in many countries. Human exposure to PFASs has been identified as a general public health concern in industrially developed countries. Food, drinking water, indoor air and contact with other contaminated media have been identified as sources of human exposure to PFASs [5,10]. Elevated PFASs in serum among populations who consume seafood frequently has been reported [5,11], and a large number of PFASs have been identified in several fish species [12,13]. 

In recent years, exposure of the general population to PFOS and PFOA has declined in developed countries such as Japan, Australia, the USA and Norway [14,15,16,17], while concentrations of perfluorononanoic acid (PFNA) and perfluorodecanoic acid (PFDA) appear to have increased in Japan and Norway. Studies on PFASs among African populations are few. To the best of our knowledge, only two studies have been carried out on maternal and umbilical blood PFAS levels in a population in South Africa [18,19]. There are scarce data and information available from populations occupationally exposed to PFASs on the African continent. 

Studies have shown higher PFAS concentrations compared to non-occupationally exposed populations among fluorochemical production workers, textile workers and professional ski waxers [20,21,22,23]. Maternal PFOA serum concentrations relating to electronic waste recycling have been surveyed in China [24]. A correlation between this specific exposure source of e-waste recycling together with involvement in e-waste with higher PFOA concentrations and adverse effects on birth outcomes was observed [24]. Thus, the main aim of the present study was to provide data on human concentration levels and distribution patterns of PFASs in an African population consisting of four groups of workers in the city of Kumasi (Ghana), and to ascertain whether or not the differences in exposure among the studied groups relate to their occupation. A further attempt was made to assess possible associations between PFASs and mercury (Hg) in whole blood (B-Hg) as a marker of seafood consumption. 

## 2. Material and Methods

### 2.1. Study Design and Subjects

Groups of male electronic repair workers (ERWs), lead battery repair workers (LBRWs) and male referents were invited to participate in this cross-sectional study. A group of female petty traders (FPTs) plying their trade within the vicinity of the workplaces of the ERWs and LBRWs was also recruited. The main inclusion criterion for selection of participants was at least one year of employment in the workplace. Participation was restricted to subjects between 18 and 50 years of age. In total, 85 ERWs working in 21 different electronic workshops in Bantama (Kumasi) were asked to participate in order to reach the target number of 64 ERWs. 

Their main job tasks were dismantling, soldering, welding and finally reassembling electronic equipment (e.g., televisions, radios, video players and computers). Eligible for inclusion as LBRWs were subjects working at two small-scale lead battery workshop sites at Suame Magazine and Asafo Fitam (Kumasi, Ghana). Altogether, 92 subjects were approached for participation in the study to reach the target of 64 LBRWs. Details have been published [25]. Seventy-nine subjects were recruited from the immediate environs of the workshops of the ERWs and LBRWs as referents, based on the assumption that they belong to the same socio-demographic level as the target groups and are not occupationally exposed to the compounds in question. Altogether, 65 referents agreed to participate. Their work included selling items such as automobile spare parts and engineering materials (excluding lead batteries) or selling (but not repairing) used electronic equipment such as televisions, radios, computers, etc. Altogether, 52 FPTs were invited into the study, of whom 26 consented to participate. 

The FPTs comprised women selling goods within the close vicinity of the working environments of the workshops where the LBRWs and ERWs were employed. Subjects with known chronic diseases, e.g., cancer or heart diseases, were excluded from the study. Likewise, known drug or alcohol abuse led to exclusion. Furthermore, since malaria is widespread in Ghana, subjects with active malaria at the time of the examinations were excluded. Ethical approval for the study was obtained from the Committee on Human Research Publication and Ethics of the School of Medical Sciences, Kwame Nkrumah University of Science and Technology/Komfo Anokye Teaching Hospital. The study was further approved by the Regional Committee for Medical Research Ethics of Northern Norway (code 2011/729). An informed written consent was obtained from all participants.

### 2.2. Examinations

Information on background variables, exposure and potential confounders (e.g., job history, medical history, alcohol consumption) of importance for the interpretation of the results were collected by the use of a self-administered questionnaire. Pregnancy-related information was recorded for the female participants. The completeness of the answers was checked by the main investigator. Biological samples were collected by authorized health staff after information of the procedure was given to the participants. Blood and first voided morning urine samples were collected on the same day.

### 2.3. Collection of Biological Samples

Whole blood was collected in 5 mL plastic vacutainer tubes with lithium-heparin (Zhejiang Kangshi Medical Devices Co., Ltd., Hangzhou China) after cleaning of the skin with deionized water and ethanol. Whole blood was also collected in 5 mL vacutainers without additives (Vacuette^®^, Greiner Labortechnik, Gmbh, Austria) for the harvesting of serum. The latter tubes were rested for 30 min after sampling and centrifuged for 10 min at 1500× *g*. Serum was pipetted off into 1.0 mL cryotubes (Sarstedt AG, Numbrecht, Germany) for long-term storage. First voided morning urine samples were collected in 10 mL Sarstedt polypropylene (PP) tubes (Sarstedt AG, Numbrecht, Germany) and transferred to 5 mL PP tubes (Greiner Bio-one, CELLSTAR^®^, Stonehouse, UK) when the participants brought their urine samples for examination. The biological samples were stored at −20 °C immediately after collection at Komfo Anokye Teaching Hospital (KATH), Kumasi, before shipment to the National Institute of Occupational Health, Norway (NIOH) for long-term storage at −20 °C.

### 2.4. Analysis of Trace Elements in Biological Samples

Blood, serum and urine samples were analyzed for selected trace elements at NIOH by inductively coupled plasma sector field mass spectrometry (ICP-SF-MS) using an Element 2 mass spectrometer (Thermo Electron, Bremen, Germany). Details have been published [25]. The limits of detection (LODs) for B-Hg and Hg and tin (Sn) in urine (U-Hg and U-Sn) were 0.13, 0.074 and 0.029 µg/L, respectively. The creatinine (cr) concentration in urine was measured with an SFA-200 flow injection analyzer (Burkard Scientific Ltd., Uxbridge, UK) according to the Jaffé reaction.

### 2.5. Analysis of PFAS in Serum

Serum samples were analyzed for PFASs at the Laboratory for Analysis of Environmental Pollutants at the University Hospital of North Norway in Tromsø (Norway) according to a fully validated high-throughput sample preparation method [26]. Briefly, samples thawed in the refrigerator overnight were brought to room temperature and sonicated prior to sample preparation on a Tecan Freedom Evo 200 automated liquid handler (Männedorf, Switzerland) equipped with an eight-channel liquid handler arm, a robotic manipulator arm and a Te-Vac station for solid phase extraction. The instrumental analysis was performed with a Waters Acquity ultra-high-pressure liquid chromatography system (UPLC, Milford, MA, USA) coupled to a Xevo TQ-S mass spectrometer (Waters, Milford, MA, USA). Electrospray ionization in negative mode (ESI^-^) was applied together with multiple reaction monitoring mode (MRM) in the tandem mass spectrometer for recording chromatograms. Altogether, 18 PFASs were quantified: perfluorobutane sulfonate (PFBS), perfluoropentane sulfonate (PFPS), perfluorohexane sulfonate (PFHxS), perfluoroheptane sulfonate (PFHpS), PFOS, perfluorononane sulfonate (PFNS), perfluorodecane sulfonate (PFDS), perfluorododecane sulfonate (PFDoDS), perfluorooctane sulfonamide (PFOSA), perfluorohexanoate (PFHxA), perfluoroheptanoate (PFHpA), PFOA, PFNA, PFDA, perfluoroundecanoate (PFUnDA), perfluorododecanoate (PFDoDA), perfluorotridecanoate (PFTrDA) and perfluorotetradecanoate (PFTeDA). The detection frequencies (DFs) and the method´s limit of detections (MLD) are presented in Supplemental Table S1. Quantitative data were obtained using Masslynx and Targetlynx software (Version 4.1, Waters, Milford, MA, USA) and achieved by the internal standard method with isotope-labeled PFAS. The linear isomers of PFSA were used for quantification of the sum concentrations and the linear and sum concentrations were used for calculating the contribution of the branched compounds.

Targetlynx software was used to calculate MLDs for each individual sample (MLD*i*) and each individual analyte with a signal to noise ratio of 3 divided by the related sample amount. Method limit of quantification (MLQ) was defined as three times the MLD. To reduce possible bias of left-censored data analyses, the actual values between MLQ and MLD were used. PFAS concentrations below the MLD were replaced by MLD*i* divided by 2. For quality assurance, four blank samples, four SRM 1957 and 1958 (NIST, Gaithersburg, MD, USA) samples and three bovine serum samples (Sigma Aldrich, Steinheim, Germany) were prepared within each batch of 96 samples. During analysis, solvent injections were done regularly in order to monitor instrumental background and carry-over effects. Differences from the assigned mean reference concentrations were between 5 and 11% in the present study. 

### 2.6. Statistical Analysis

Statistical data treatment was performed only for PFASs with detection rates ≥ 80%. PFASs with detection rates < 80% were included in the sum concentrations (Σ-PFCA, Σ-PFSA and Σ-PFAS). The distribution of the variables was visually assessed, and their skewness calculated. Variables with skewness exceeding 2.0 were log-transformed. Thus, since B-Hg, U-Hg, U-Sn, L-PFHxS, Br-PFHxS and Σ-PFHxS were skewed, they were log-transformed. The geometric means (GMs) and ranges are presented for these variables, while arithmetic means (AMs) and ranges are given for the others. Differences between groups were assessed using ANOVA, and the least square difference was calculated to assess which groups differed from each other. A general linear model was applied to adjust for relevant covariates between groups. Multiple linear regression analysis (backward procedure) was carried out with PFASs in serum as dependent variables. The main independent variables were B-Hg (lg), being an ERW (1/0), sex (1/0), age (years) and body mass index (BMI) (kg/m^2^). Two-tailed *p*-values < 0.05 were considered to be of statistical significance. The statistical data package SPSS^®^, version 25.0 (IBM Corp., Armunk, NY, USA), was used for the statistics.

## 3. Results and Discussion

### 3.1. Background Data, Hg and Sn

Background data of the participants and results of the measurements of Hg and Sn in biological samples are shown in Table 1 for each of the four groups. Age was similar in the groups, while the FPTs had higher BMI. The ERWs had statistically significantly higher U-Sn than the referents. The B-Hg concentrations were similar in the four groups, but the LBRWs had statistically significantly lower concentrations than the referents. The U-Hg concentrations were also low.

### 3.2. Concentration and Distribution Patterns of PFASs

The analytes with an overall detection frequency (DF) > 80%, as presented in Table 2, were selected for further statistical data treatment. These data are presented as boxplots according to analyte groups of PFSA and PFCA in Supplemental Figures (Figure S1A–C). PFHpA, PFDoDA, PFTriDA and PFBS were detected in frequencies < 30% and generally in lower concentrations, while PFTeDA, PFPS, PFNSs, PFDS, PFDoDS and PFOSAs were not detected at all (Supplemental Table S1). These PFASs were not considered further. The concentrations of Σ-PFSA were approximately three to four times higher than the Σ-PFCA (Table 2). 

This is in accordance with results from a previous study on maternal serum from South Africa [18] and studies performed in urban areas of other continents [27]. The most abundant PFASs were Σ-PFOS and Σ-PFHxS (Table 2). The PFAS concentrations were generally lower in females (FPTs) compared to the male groups (ERWs, LBRWs and referents). Multiple linear regression analysis showed that age was significantly associated with most of the investigated PFASs, except Σ-PFHxS, Br-PFHxS and PFUnDA (Table 3). 

Several of the concentrations were also associated with sex, indicating higher concentrations in men. A gender-related difference in PFAS distribution was previously reported within a Chinese population group [27]. Females seem to have a tendency for a faster elimination of PFOS, PFOA and PFHxS compared to males due to blood loss via the monthly menstruation cycle [28,29,30]. The mother–child transfer of PFASs during pregnancy and breast feeding, as well as number of parity, also have to be taken into account for women who have been pregnant and given birth [12,29,31,32]. We have, however, no such data for the FPTs. To our best knowledge, there are two published studies on PFAS concentrations in humans available from the African continent [18,19].

**Figure 1:**
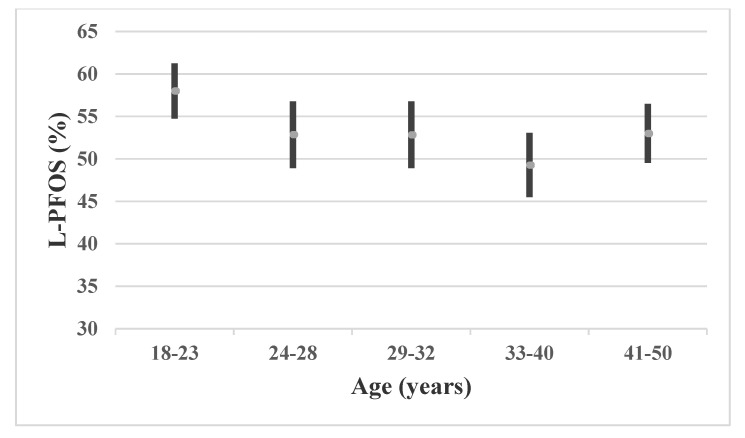
The arithmetic mean (and 95% CI) percentage of L-PFOS in serum concentrations among 193 men according to age.

PFOS concentration ranges from urban and industrial locations in South Africa were similar, while the PFOS concentrations were lower in Tanzania compared to the present study. Higher PFOA concentrations were observed in several locations in South Africa. Participants from the present study had generally higher Σ-PFHxS concentrations (range: 0.23–7.09 ng/mL) than those from South Africa (range: 0.16–3.2 ng/mL), indicating different sources of exposure. Comparing the PFAS concentrations measured in the present study with studies from non-African non-occupationally exposed populations, generally, low concentrations of PFASs were detected. Gribble et al. [33] studied PFAS serum concentrations in Gullah African Americans between 2003 and 2013 and found a significant decline for most of the investigated PFAS compounds. However, the concentrations measured in 2013 were still higher compared to the present study, which may mostly be related to differences in lifestyle and dietary habits. Serum concentrations of Australians were also higher for individual PFASs (i.e., PFOA, PFNA, PFDcA, PFHxS and PFOS) [34]. Similar observations were found in studies from New Zealand [35] and China [36], with the exception of PFHxS, where concentrations of the present study were similar to males from New Zealand. PFOS also showed lower GM concentrations in Henan, China [36]. The subjects investigated in the present study show generally lower PFAS concentrations than cohorts studied in industrialized countries. To date, there is no information available on environmental sources of PFAS in Ghana. However, it is likely that PFAS exposure sources are primarily through water and food but contact with consumer products may also contribute [37]. The mean (and 95% CI) percentages of L-PFOS, L-PFHxS and L-PFHpS related to the sum of these components among all participants were 54% (53–56), 38% (35–41) and 76% (75–77), respectively (not tabulated). Generally, it was observed that men had a lower percentage of L-PFOS (53%; 52–55) compared to female participants (62%: 58–66) (*p* < 0.001). This is in the lower end of that previously reported in humans (53–80%) [38]. Differences in metabolism could be a reason for this gender difference of the branched and linear species distribution of PFOS, but also parity (number of children/pregnancies) for the women, where they transfer a part of their PFAS burden to their children. How and in which content PFOS species (linear and the different branched forms) are affected by this transfer is unknown at this time. There was also a negative association between the percentage of L-PFOS and age (Pearson’s r = −0.20; *p* = 0.006). Stratifying the male participants into five groups indicated that the youngest male participants had higher percentages of L-PFOS than the other age groups (Figure 1). This may suggest that younger participants have been less exposed to Br-PFOS than older participants, which may be explained by a shift in production with ECF to more modern methods of telomerization. Any shift in isomer pattern in contaminated fish or other sources of exposure compared to previous results remains to be elucidated. 

### 3.3. PFASs and Sn in ERWs

**Figure 2:**
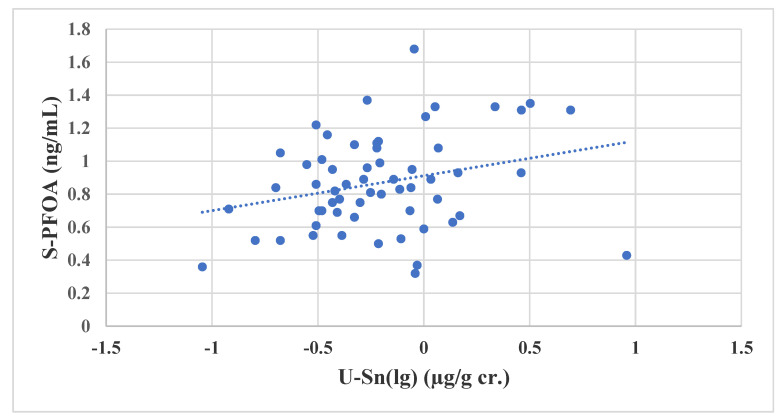
The association between PFOA in serum and U-Sn (lg) among 64 electronic repair workers.

The ERWs had higher concentrations of serum PFOA and U-Sn than the other groups. The concentrations of U-Sn (lg) and PFOA in serum of the ERWs were statistically significantly associated (Pearson’s r = 0.28; *p* = 0.035) (Figure 2). Years of exposure as an ERW and age were not associated with U-Sn (lg) or PFOA. Solders used for the repair of electronic equipment contain tin [39]. Since it is known that fluoropolymers are used in memory cards in electronic equipment [3], it may be reasonable to assume that, e.g., PFOA evaporates during soldering processes at temperatures that will not cause thermal degradation of PFOA [40]. This assumption may be plausible because of the statistically significant association between serum PFOA and U-Sn. Another possible exposure route of Sn and PFOS among the electronic repair workers is personal hygiene due to contamination of fingers, etc. [41]. However, when using PFOA as a dependent variable in the ERWs, B-Hg (lg) was also associated with PFOA, together with U-Sn (lg) (PFOA = 0.69 + 0.24U-Sn (lg) (*p* = 0.02) + 0.39 B-Hg(lg) (*p* = 0.02)) (not tabulated). 

This could indicate that PFOA concentrations among ERWs are associated with dietary intake and occupational exposure. We have not found any previous reports on PFOA in ERWs. However, one study from China reported higher PFOA concentrations in maternal serum samples of women living near an electronic waste recycling facility [24]. That study showed evidence for an association between involvement in e-waste recycling and higher PFOA serum concentrations, which is compatible with the observations in our study. 

Although the PFOA concentrations are statistically significantly higher in the group of ERWs, the concentrations are low compared to other occupationally exposed groups, such as fluoropolymer industry workers or professional ski-waxers [20,21]. There is also paucity of data on U-Sn in occupationally inorganic Sn-exposed populations, but previous studies have shown increased U-Sn in exposed workers [42]. No association was observed between U-Sn (lg) and Br-PFHxS (lg) or Σ-PFHxS (lg). 

### 3.4. Associations between Biomarkers of Seafood Consumption and PFASs

**Figure 3:**
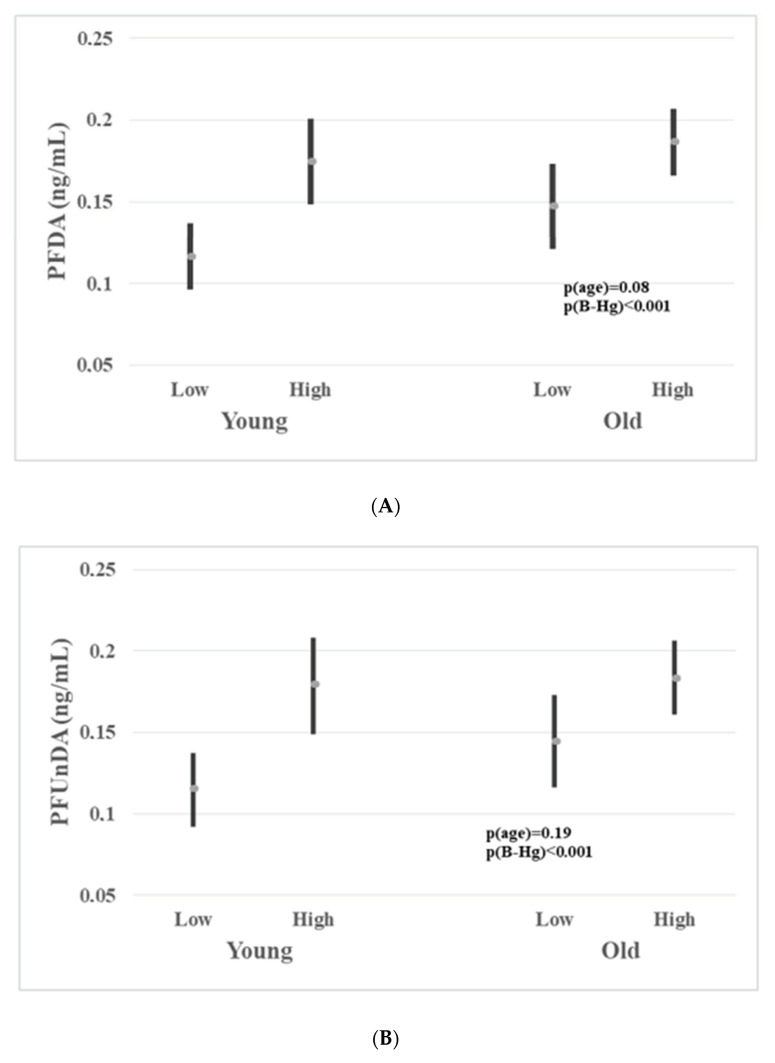

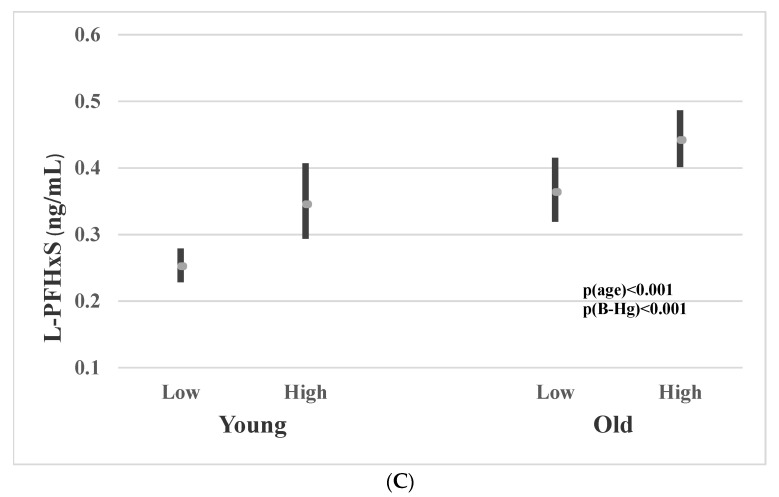
(**A**–**C**) comprises three panels: PFDA (**A**), PFUnDA (**B**) and L-PFHxS (**C**). The arithmetic mean (and 95% CI) serum concentrations of PFDA, PFUnDA and L-PFHxS in male participants stratified into two equally large groups of young (<30 years of age) and old (≥30 years of age) participants according to the concentrations of B-Hg stratified into to equally large groups of low (B-Hg 1.0–3.7 µg/L) and high (B-Hg 3.8–17.9 µg/L) concentrations.

**Figure 4:**
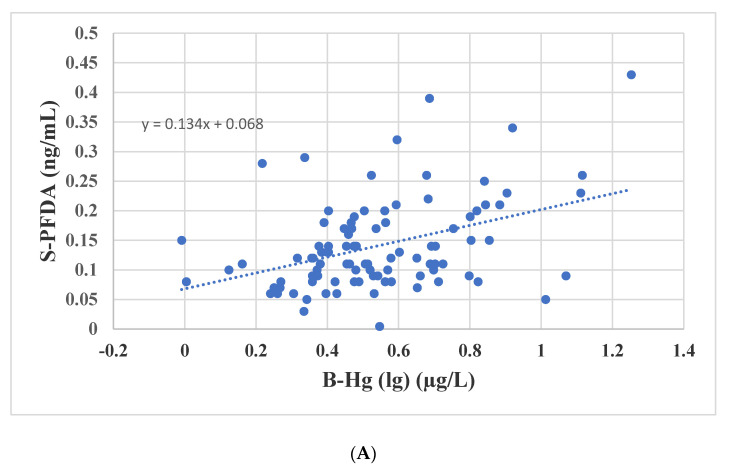

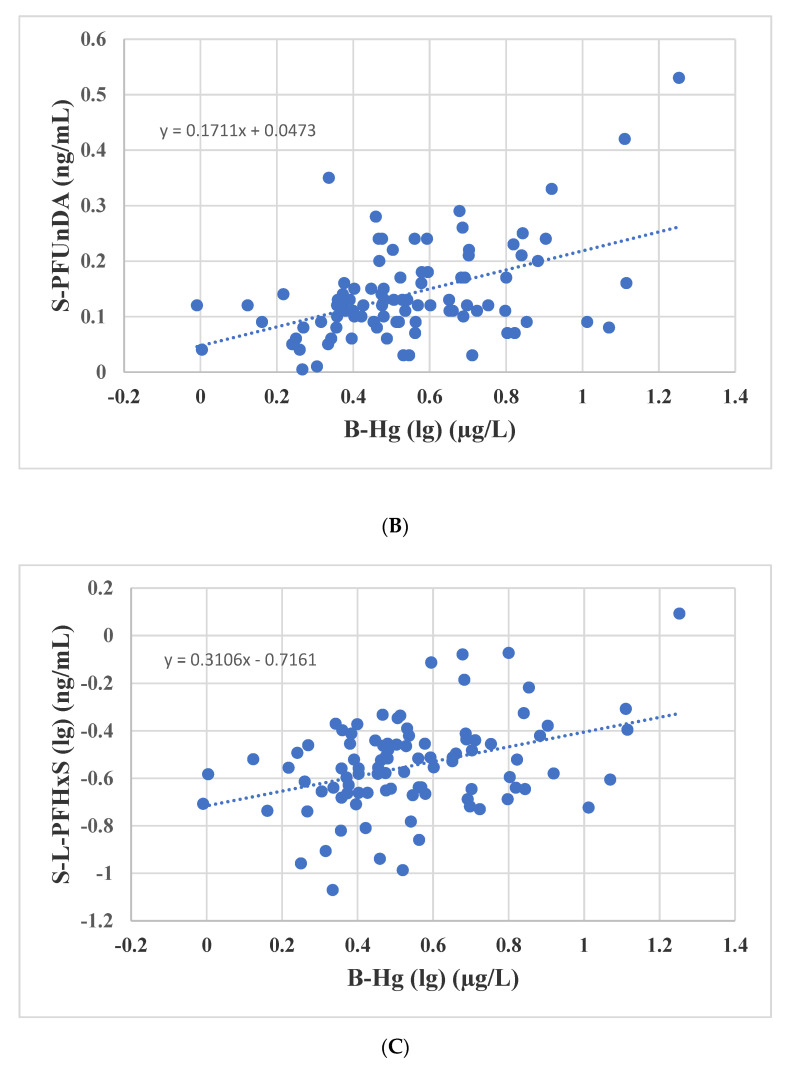
(**A**) The association between PFDA in serum and B-Hg in 95 men below 30 years of age. (**B**) The association between PFUnDA in serum and B-Hg in 95 men below 30 years of age. (**C**) The association between L-PFHxS in serum and B-Hg in 95 men below 30 years of age.

Multiple linear regression analysis showed that B-Hg and sex were strong predictors for many PFAS concentrations (Table 3). In order to exclude the effect of sex, only male participants were further studied when assessing associations between PFAS and B-Hg. The male participants were stratified into two equally large groups according to age (AM 24.7; 18–29 years and AM 38.3; 30–50 years) and B-Hg (GM 2.6; 1.0–3.7 µg/L and GM 5.7; 3.8–17.9 µg/L). Figure 3A–C show the concentrations for those PFASs that were statistically significantly different according to “low” and “high” B-Hg in both age strata. These PFASs were PFDA (Figure 3A), PFUnDA (Figure 3B) and L-PFHxS (Figure 3C). The univariate associations between B-Hg (lg) and PFDA, PFUnDA and L-PFHxS (lg), respectively, are of statistical significance in both age strata. Figure 4A–C show the univariate associations between B-Hg (lg) and PFDA (Figure 4A), PFUnDA (Figure 4B) and L-PFHxS (Figure 4C), respectively, in the group of male participants of 18–29 years of age. It is noteworthy that L-PFHxS was statistically significantly associated with B-Hg(lg), while no association was observed between Br-PFHxS (lg) and B-Hg (lg) (Table 3).

Women also showed some relationship between PFASs and B-Hg. For example, statistically significant associations between B-Hg (lg) and PFNA (Pearson’s r = 0.45; *p* = 0.03), L-PFOS (r = 0.45; *p* = 0.02) and Σ-PFOS (r = 0.45; *p* = 0.02), respectively, were observed among the FPTs, while the association with PFDA (r = 0.37; *p* = 0.07) was nearly statistically significant. Although available data indicate an association between B-Hg (lg) and some of the PFASs among the FPTs, the sample size of 25 women is too small to perform an age-stratified analysis. In addition, the lack of information on reproductive factors such as pregnancy and breastfeeding, parity and monthly menstrual loss makes it more difficult to study the association between PFASs and B-Hg among women. Thus, a substantially larger group of women would be required to more completely investigate sex differences. 

The mean per capita annual fish consumption in Ghana has been estimated to be around 26 kg [43]. According to the FAO [44], fish accounts for as much as 60 percent of animal protein in the average Ghanaian diet, and 22.4 percent of household food expenditures. Studies in Ghana have shown a mean Hg concentration up to 0.187 µg/g fish muscle tissue, depending on fish species and the location where the fish is caught [45,46]. These concentrations are well below the European Union recommendation for Hg in fish for human consumption (<0.5 mg/kg). To the best of our knowledge, PFAS content of fish has not been determined in Ghana. We used the individual concentrations of B-Hg as a biomarker for seafood consumption. As inorganic Hg is excreted in urine, in contrast to only minor amounts of organic Hg, the low U-Hg concentration in this population indicates negligible exposure to inorganic Hg compounds [42]. Thus, B-Hg may be regarded as a reasonable biomarker for fish consumption [42]. The regression analyses showed that B-Hg was a stronger predictor of PFAS concentrations in serum than age. The results also suggested that the associations between B-Hg and PFCA were stronger than between B-Hg and PFSA. All PFAS concentrations detected in serum of the study participants were positively associated with the concentrations of B-Hg, with the exception of Σ-PFHxS and Br-PFHxS (Table 3). This indicates that consumption of seafood is related to the measured concentrations. It is in this context of interest that a recent experimental study of carp showed high accumulation of PFDoDA, PFDA and PFUnDA, while there was almost no PFHxS accumulation [47]. However, that study also measured PFHxS isomers and showed that around 90% of the accumulated PFHxS was in the linear form, supporting the association between B-Hg (lg) and L-PFHxS that we observed in the present study.

The strongest associations in this study were observed between B-Hg and PFDA and PFUnDA (Figure 4A,B), respectively. Subjects with “high” B-Hg had statistically significantly higher PFUnDA and PFDA than subjects with “low” B-Hg in the respective age strata. The subjects with “low” B-Hg had B-Hg concentrations from 1.0 to 3.7 µg/L, indicating that they also had a significant seafood consumption. The concentration of Hg in hair, another well-known biomarker of organic Hg intake, was highly associated with serum PFUnDA and PFDA in children [11]. That study also reported statistically significant associations between hair Hg and PFHpS, PFOS and PFNA, which is compatible with our observations. Increasing serum concentrations of PFNA, PFDA, PFUnDA and PFOS related to seafood consumption were also observed in a recent study [48,49]. 

Studies of different fish species or food baskets have shown that many different PFSAs may be present in seafood [13,48,50]. Some of the PFSA concentrations were statistically significantly lower among the LBRWs as compared to the referents. Generally, these statistically significant differences disappeared when adjusting for B-Hg, indicating that differences in fish consumption between the groups may be the cause for the lower concentrations among the LBRWs.

## 4. Conclusions

For the first time, an assessment of internal exposure concentrations of PFASs in selected groups of workers from Ghana is presented. The PFAS concentrations were comparable with other populations with a similar degree of urbanization, and lower compared to countries with higher industrial activity. A positive association with age was shown for most of the PFASs. Female petty traders had generally lower PFAS serum concentrations compared to the male groups. Electronic repair workers had higher serum PFOA concentrations, which is compatible with the known use of PFASs in electronic equipment. The concentration of B-Hg had a substantial impact on several PFAS concentrations, indicating that seafood may be an important source of intake. Hence, a broader monitoring of PFAS concentrations within the adult population and different fish species in Ghana may be warranted.

## Figures and Tables

**Table 1:** Background data of lead battery repair workers (LBRWs), electronic repair workers (ERWs), referents and female petty traders (FPTs) under study.

	Referents (N = 65)	ERWs (N = 64)	LBRWs (N = 64)	FPTs (N = 25)	
	AM * (Min–Max)	AM (Min–Max)	AM (Min–Max)	AM (Min–Max)	
p_ANOVA_					
Age (years.) ^c^	30.2 (18–50)	32.6 (18–50)	31.8 (20–49)	34.2 (20–49)	0.17
BMI (kg/m^2^) ^c^	23.0 (16.5–31.5)	22.8 (17.9–29.4)	23.3 (17.3–31.1)	28.2 (16.2–40.0)	<0.001
Work-years ^b^	8.9 (1–30)	10.8 (2–30)	11.4 (1–30)	6.2 (1–20)	0.007
Smokers (in %)	1.6	0	3.1	0	Na ^§^
Alcohol users (in %)	20.3	23.4	15.6	4.0	na
U-Sn (µg/g. cr.) ^†a^	0.32 (<LOD ^‡^–8.1)	0.62 (0.09–9.1)	0.24 (0.06–8.1)	0.36 (0.11–1.9)	<0.01
U-Hg (µg/g cr.) ^†c^	0.26 (<LOD–1.2)	0.27 (<LOD–2.6)	0.22 (<LOD–4.2)	0.42 (<LOD–6.9)	0.05
B-Hg (µg/L) ^†b^	4.3 (1.5–18)	3.6 (1.3–13)	3.6 (1.0–9.3)	3.6 (2.1–15)	0.14

* arithmetic mean; ^†^ geometric mean; ^a^ *p* < 0.05 between referents and ERWs; ^b^ *p* < 0.05 between referents and LBRWs; ^c^ *p* < 0.05 between referents and FPTs; ^§^ not applicable; ^‡^ limit of detection.

**Table 2:** Arithmetic mean (AM) (and min–max) concentrations (ng/mL) of perfluoroalkyl substances in serum of referents, electronic repair workers (ERWs), lead battery repair workers (LBRWs) and female petty traders (FPTs).

	Referents ^1^	ERWs	LBRWs	FPTs	
	(N = 65)	(N = 64)	(N = 64)	(N = 25)	
	AM (Min–Max)	AM (Min–Max)	AM (Min–Max)	AM (Min–Max)	p_ANOVA_
PFOA ^abc^	0.66 (0.24–1.4)	0.87 (0.32–1.7)	0.52 (0.16–0.91)	0.40 (0.17–0.61)	<0.001
PFNA ^bc^	0.29 (0.11–0.84)	0.28 (0.07–0.56)	0.25 (0.08–0.55)	0.24 (0.10–0.49)	0.07
PFDA	0.17 (0.01–0.43)	0.15 (0.05–0.39)	0.15 (0.03–0.40)	0.16 (0.05–0.33)	0.58
PFUnDA	0.17 (<MLD ^‡^–0.53)	0.15 (0.03–0.42)	0.14 (<MLD–0.39)	0.14 (0.06–0.33)	0.20
Σ-PFCA ^abc^	1.3 (0.39–3.3)	1.5 (0.60–2.3)	1.1 (0.50–1.9)	0.95 (0.40–1.7)	<0.001
L-PFHxS ^†c^	0.35 (0.09–1.2)	0.37 (0.12–1.1)	0.31 (0.10–0.83)	0.21 (0.07–3.4)	<0.001
Br-PFHxS ^†a^	0.49 (0.01–4.1)	0.78 (0.07–5.3)	0.65 (0.09–5.5)	0.44 (0.09–6.9)	0.02
Σ-PFHxS ^†a^	0.99 (0.35–4.2)	1.3 (0.25–6.1)	1.1 (0.25–5.8)	0.75 (0.23–7.1)	0.008
L-PFHpS ^c^	0.08 (<MLD–0.28)	0.08 (<MLD–0.16)	0.08 (0.04–0.19)	0.02 (<MLD–0.12)	<0.001
Br-PFHpS ^c^	0.02 (<MLD–0.06)	0.03 (<MLD–0.06)	0.02 (<MLD–0.04)	0.01 (<MLD–0.04)	<0.001
Σ-PFHpS ^c^	0.10 (<MLD–0.32)	0.11 (<MLD–0.21)	0.10 (0.05–0.22)	0.03 (<MLD–0.16)	<0.001
L-PFOS ^b^	1.8 (0.33–5.3)	1.7 (0.49–4.4)	1.4 (0.20–3.6)	1.4 (0.53–3.3)	0.04
Br-PFOS ^c^	1.4 (0.43–3.6)	1.4 (0.37–3.3)	1.2 (0.46–2.9)	0.80 (0.29–1.7)	<0.001
Σ-PFOS ^bc^	3.2 (0.88–7.4)	3.2 (1.1–6.6)	2.7 (0.77–5.6)	2.2 (0.82–4.5)	0.002
Σ-PFSA ^c^	4.5 (1.4–10.1)	4.9 (1.7–11.5)	4.1 (1.7–8.0)	3.4 (1.2–9.3)	0.003

^1^ one subject missing; ^†^ geometric mean (GM); ^a^ *p* < 0.05 between referents and ERWs; ^b^ *p* < 0.05 between referents and LBRWs; ^c^ <0.05 between referents and FPTs; ^‡^ method’s limit of detection.

**Table 3:** Results from multiple linear regression analysis of perfluoroalkyl substance (PFAS) serum concentrations (ng/mL) as dependent variables. Independent variables are being an electronic repair worker (ERW (1/0), blood mercury concentration ((B-Hg (lg)), age, body mass index (BMI) and sex (1/0). β-coefficients and multiple r are presented.

	ERW	B-Hg (lg)	Age	BMI	Sex	Multipler
PFOA	0.29 ***	0.32 ***	-	-	0.18 **	0.59 ***
PFNA	-	0.19 ***	0.002 *	−0.004 *	-	0.43 ***
PFDA	-	0.13 ***	0.001 *	-	-	0.40 ***
PFUnDA	-	0.14 ***	-	-	-	0.35 ***
L-PFHxS (lg)	-	0.26 ***	0.008 ***	-	0.22 ***	0.53 ***
Br-PFHxS (lg)	0.16 *	-	-	-	-	0.17 *
Σ-PFHxS (lg)	0.09 *	-	-	-	0.14 *	0.23 **
L-PFHpS	-	0.05 ***	0.001 ***	-	0.06 ***	0.59 ***
Br-PFHpS	0.005 *	0.01 **	0.001 ***	-	0.02 ***	0.55 ***
Σ-PFHpS	-	0.06 ***	0.002 ***	-	0.08 ***	0.60 ***
L-PFOS	-	1.2 ***	0.02 *	−0.04 **	-	0.37 ***
Br-PFOS	-	0.79 ***	0.02 ***	-	0.57 ***	0.54 ***
Σ-PFOS	-	1.9 ***	0.03 **	-	0.83 **	0.47 ***

* *p* < 0.05; ** *p* < 0.01; *** *p* < 0.001.

## Data Availability

The data presented in this study are available on request from authors.

## Supplementary Materials

The following are available online at https://www.mdpi.com/1660-4601/18/4/1581/s1, Table S1: Detection frequencies (DF) and method’s limit of detections (MDL) of PFAS (ng/mL) in serum determined among all participants (N = 218). Only values > MDL have been considered in the descriptive statistics, and 90th percentiles have only been calculated when DF was larger than 80%; Figure S1: Boxplots of selected PFASs (ng/mL) with a detection frequency >80% for lead battery repair workers (LBRWs), electronic repair workers (ERWs) and referents. For female petty traders (FPTs) the PFHpSs had a lower detection frequency (24%). Panel A: selected PFCAs; Panel B: selected PFSAs; Panel C: sum concentrations of PFCAs, PFSAs and PFAS.
