# Supplementary material for: Per- and Polyfluoroalkyl Substances in Human Serum Samples of Selected Populations from Ghana
Source: Int J Environ Res Public Health. 2021 Feb 8;18(4):1581. doi: 10.3390/ijerph18041581 (PMC7914835; doi:10.3390/ijerph18041581)

Supplemental tables and figures

Table S1 Detection frequencies (DF) and method's limit of detections (MDL) of PFAS (ng/mL) in serum determined among all participants (N=218). Only values > MDL have been considered in the descriptive statistics, and 90th percentiles have only been calculated when DF was larger than 80%.

	DF (%)	MDL	Concentrations<br>Median	90th percentile	min-max
**PFHxA**	0	0.008-0.094	-	-	-
**PFHpA**	17	0.007-0.032	0.04	-	0.02-0.11
**PFOA**	100	0.049-0.080	0.59	1.1	0.16-1.7
**PFNA**	100	0.005-0.034	0.25	0.40	0.07-0.84
**PFDcA**	100	0.002-0.053	0.14	0.27	0.03-0.43
**PFUnDA**	99	0.002-0.044	0.13	0.27	0.03-0.53
**PFDoDA**	13	0.001-0.044	0.04	-	0.02-0.12
**PFTriA**	21	0.001-0.041	0.05	-	0.02-0.12
**PFTeA**	0	0.009-0.57	-	-	-
**PFBS**	10	0.001-0.007	0.02	-	0.01-0.05
**PFPS**	0	0.002-0.024	-	-	-
**L-PFHxS**	100	0.005-0.055	0.34	0.59	0.07-3.4
**Σ-PFHxS**	100	0.005-0.055	0.98	2.8	0.23-7.1
**L-PFHpS**	83	0.002-0.019	0.08	0.13	0.03-0.28
**Σ-PFHpS**	83	0.002-0.019	0.11	0.16	0.04-0.32
**L-PFOS**	100	0.001-0.18	1.4	2.8	0.20-5.3
**Σ-PFOS**	100	0.001-0.18	2.6	4.8	0.77-7.4
**L-PFNS**	0	0.001-0.023	-	-	-
**Σ-PFNS**	0	0.001-0.023	-	-	-
**L-PFDcS**	0	0.001-0.041	-	-	-
**Σ-PFDcS**	0	0.001-0.041	-	-	-
**PFDoS**	0	0.001-0.016	-	-	-
**L-PFOSA**	0	0.005-0.008	-	-	-
**Σ-PFOSA**	0	0.005-0.008	-	-	-

Figure S1 A-C Boxplots of selected PFASs (ng/mL) with a detection frequency >80% for lead battery repair workers (LBRWs), electronic repair workers (ERWs) and referents. For female petty traders (FPTs) the PFHpSs had a lower detection frequency (24%). Panel A: selected PFCAs; Panel B: selected PFSAs; Panel C: sum concentrations of PFCAs, PFSAs and PFAS.

Panel A

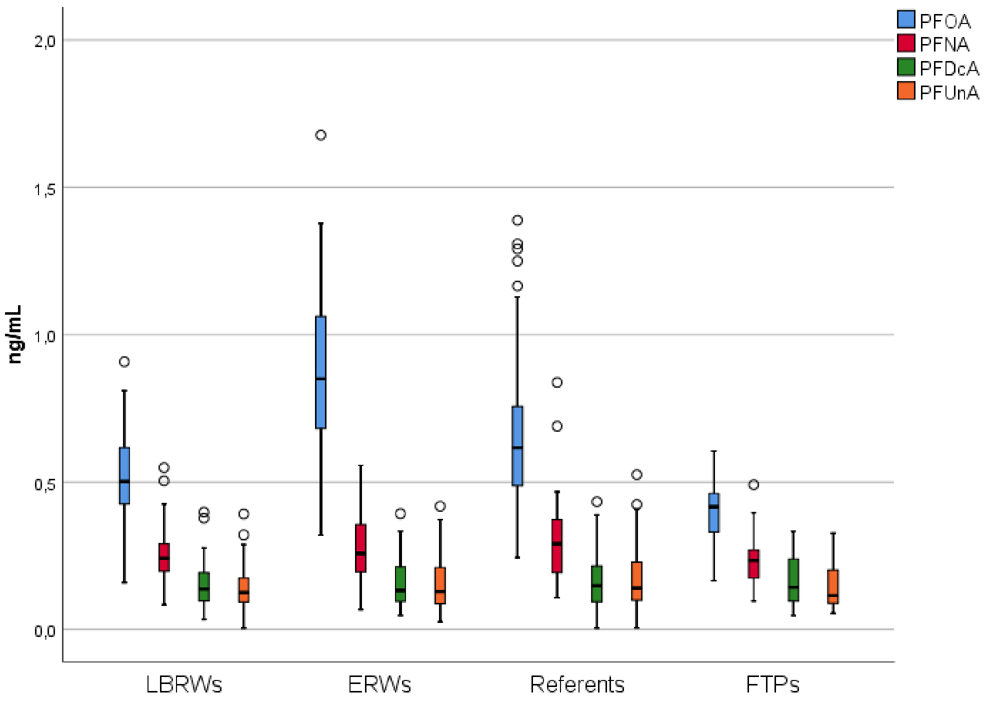

PFOA
PFNA
PFDcA
PFUnA
ng/mL
2,0
1,5
1,0
0,5
0,0
LBRWs
ERWs
Referents
FTPs

Panel B

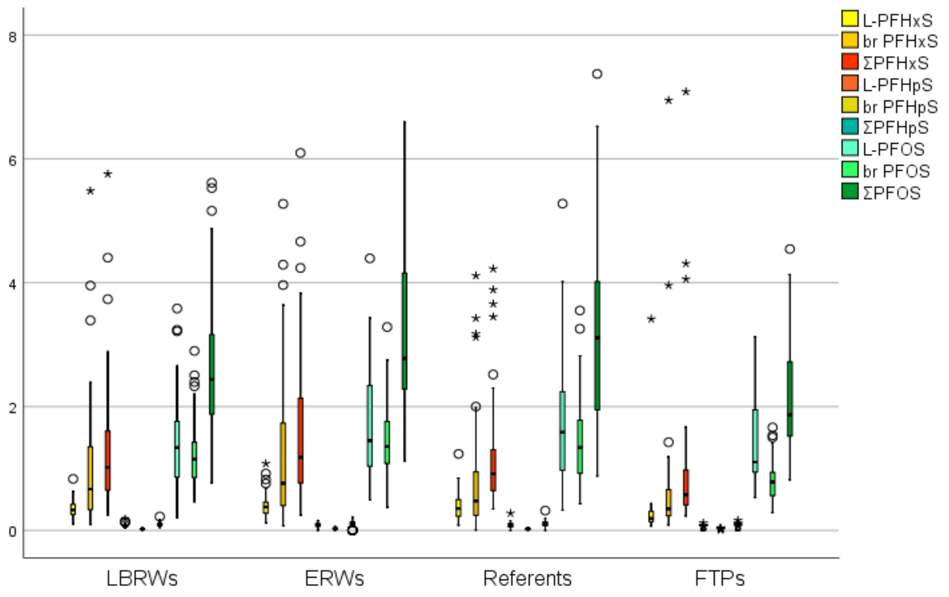

Panel C

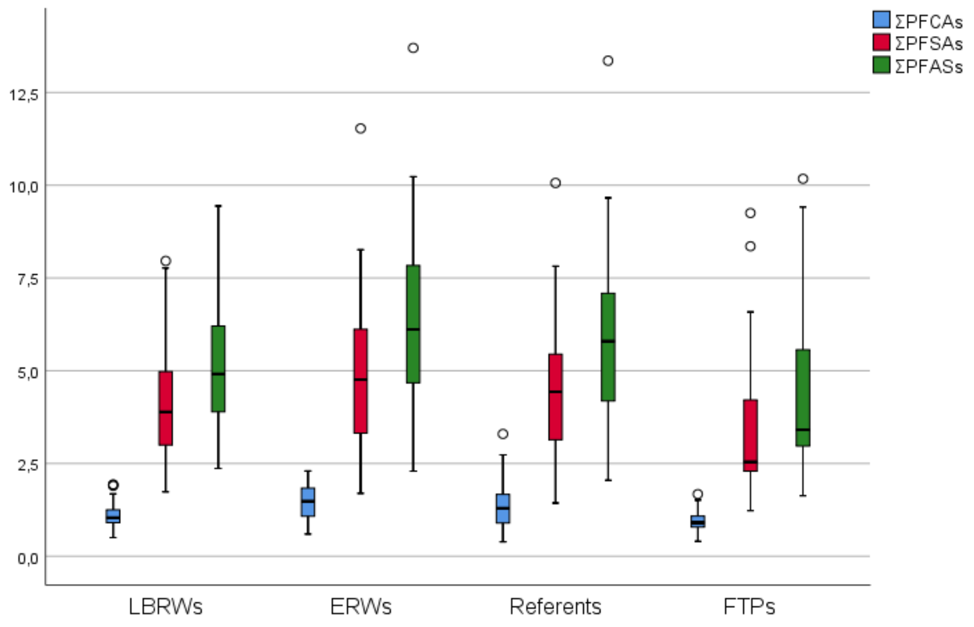

Supplement: Supplementary file 1 [file ijerph-18-01581-s001.pdf]
